# Analysis of multiple factors influencing the survival of patients with advanced gastric cancer

**DOI:** 10.18632/aging.205820

**Published:** 2024-05-13

**Authors:** Xinqiang Zhu, Beibei Ge, Linchun Wen, Hailong Huang, Xiaohong Shi

**Affiliations:** 1 The Affiliated Suqian Hospital of Xuzhou Medical University, Suqian, Jiangsu 223800, PR China; 2 Affiliated Hospital of Jiangsu University, Zhenjiang, Jiangsu 212000, PR China

**Keywords:** multiple factors, survival, advanced gastric cancer

## Abstract

Objective: The aim of this study was to investigate the main factors influencing the survival of patients with advanced gastric cancer.

Methods: The clinicopathological data of 120 patients with advanced gastric cancer were analyzed retrospectively, and clinical and pathological data were collected. Tumor tissue staging and grading were re-evaluated, and 5-year overall survival was followed up. The classified data were described by percentages, and the continuous data were described by standard deviations or medians. Univariate analysis was performed using the χ^2^ test or rank-sum test, followed by Kaplan-Meier survival analysis to calculate the median survival time and 5-year cumulative survival. A multivariate Cox regression model was used to evaluate the independent risk factors affecting survival. The test level was α = 0.05.

Results: Patients were followed up for 0 to 60 months, the 5-year overall survival rate was 36.2%, and the median survival time was 53.0 ± 1.461 months. K-M and log-rank test results revealed that tumor location, degree of differentiation, depth of invasion, regional lymph node involvement, and postoperative tumor stage were correlated with a decreased 5-year survival rate (*P* < 0.05). A multivariate Cox risk regression model was used to analyze the degree of histological differentiation (HR = 1.441; 95% CI = 1.049–1.979; *P* = 0.024), regional lymph node (HR = 1.626; 95% CI = 1.160–2.279; *P* = 0.005), and pTNM stage (HR = 2.266; 95% CI = 1.335-3.847; *P* = 0.002), which are independent risk factors for poor survival. Tumor location (*P* = 0.191), invasion depth (*P* = 0.579) and tumor size (*P* = 0.324) were not found to be independent risk factors.

Conclusion: The degree of tumor differentiation, regional lymph node metastasis and postoperative pathological stage were found to be independent risk factors for 5-year overall survival in patients with advanced gastric cancer. Standardized and reasonable lymph node dissection and accurate postoperative pathological staging were very important.

## INTRODUCTION

Gastric cancer is one of the most prevalent malignant neoplasms affecting the gastrointestinal system [1] and ranks as the fifth most common malignancy worldwide, following lung, breast, colorectal, and prostate cancers [2]. The current global ranking of gastric cancer-related deaths is fourth. Despite a worldwide decrease in the incidence of gastric cancer over the past century, it remains a significant contributor to mortality on a global scale. [3], and identifying prognostic factors may help predict and improve the prognosis of patients with gastric cancer. The current consensus is that surgery remains the primary curative approach for gastric cancer. With advancements in surgical technology and the integration of conventional radiotherapy, chemotherapy, and neoadjuvant therapy, the 5-year survival rate for patients with early-stage gastric cancer can reach an impressive 95% [1], while the 5-year survival rate of patients with advanced gastric cancer is a mere 20–60% [4]. Retrospective analysis of clinicopathological data from 120 patients with advanced gastric cancer admitted from 2014 to 2016 at Suqian Hospital Affiliated with Xuzhou Medical University was performed. Five-year survival was followed up, and the independent risk factors affecting 5-year survival were identified. This technique provides a strong basis for accurate evaluation and prognosis of advanced gastric cancer patients after radical resection.

## Patients and Methods

A retrospective analysis was conducted on 120 patients with advanced gastric cancer who were admitted to Suqian Hospital Affiliated to Xuzhou Medical University from 2014 to 2016. The inclusion criteria for patients were as follows: (1) r Complete pre - and post-operative follow-up data were available for gastric cancer; (2) underwent open D2 radical surgical treatment and were pathologically confirmed to have advanced gastric cancer; and (3) had all postoperative adjuvant chemotherapy regimens containing platinum/paclitaxel combined with fluorouracil.

Exclusion criteria for patients were as follows: (1) had early-stage gastric carcinoma, (2) had a surgical management of gastric carcinoma, and (3) had undergone neoadjuvant chemotherapy before surgery but no adjuvant chemotherapy after surgery; (4) the tumor was located at two or more sites; (5) had a previous history of malignant tumor; (6) had metastases of other tissues and organs.

The clinicopathological features were obtained from all original medical records as follows: age, sex, CEA level, tumor location, tumor differentiation grade, invasion depth, tumor size, nerve invasion, the presence of lymph node metastasis, and postoperative pathological stage.

The age range was 31–79 years. In total, 31 tumors were located in the upper region (25.8%), 68 tumors were located in the lower region (56.7%), and 21 tumors were located in the middle region (17.5%). Three differentiation grades were used for classification: 15 (12.5%) were highly differentiated (G1); 43 (35.8%) were moderately differentiated (G2); and 62 (51.7%) were poorly differentiated (G3%). The preoperative nutritional status of the patients was divided into nutritional risk (4 patients) and no nutritional risk (116 patients) according to the nutritional risk screening 2002 (NRS 2002) [5]. All patients had diabetes mellitus (33 patients), hypertension (49 patients) and coronary heart disease (15 patients). According to the American Society of Anesthesiologists (ASA), there were 102 patients with ASA1 and 18 patients with ASA2 (Table 1).

**Table 1:** Characteristics of relevant clinical data.

**Parameter**	**Case (*n* = 120)**	**Percentage (%)**
Age		
<65 years	49	40.8
≥65 years	71	59.2
Mean ± standard deviation	52.35 ± 2.168	
Median	63.5	
Age range	31–79	
Sex		
Female	56	46.7
Male	64	53.3
CEA level		
Rise	44	36.7
Normal	76	63.3
Tumor location		
Proximal gastric cancer	31	25.8
Central Gastric cancer	21	17.5
Distal gastric cancer	68	56.7
Tumor differentiation		
G1	15	12.5
G2	43	35.8
G3	62	51.7
Invasion depth		
T2	51	42.5
T3/T4	69	57.5
Tumor size		
<5 cm	76	63.3
≥5 cm	44	36.7
Regional lymph nodes		
N0	31	14.6
N1	58	27.4
N2	79	37.3
N3	44	20.7
Lymphatic carcinoma thrombus		
No	75	62.5
Yes	45	37.5
Nerve invasion		
No	87	72.5
Yes	33	27.5
pTNM stages		
Ib	29	24.2
II	35	29.2
III	56	46.6
Nutritional status		
Nutritional risk	4	3.3
No nutritional risk	116	96.7
Concomitant disease		
Diabetes	33	27.5
Hypertension	49	40.8
Coronary heart disease	15	12.5
ASA classification		
ASA1	102	85.0
ASA2	18	15.0
Postoperative complication		
Anastomotic leak	5	4.20
Anastomotic hemorrhage	3	2.50
Pulmonary infection	3	2.50
Incision infection	6	5.0

### Follow-up

The follow-up of 120 patients was conducted over a period of 60 months through telephone, text message or outpatient visits. The observation time started at discharge after the hospital operation and ended at 5 years. The outcomes included survival, death and loss to follow-up. Overall survival (OS) was defined as the time span between surgery and death.

### Statistical methods

The statistical analysis was conducted using SPSS 19.0 software. The categorical data are presented as percentages, and the description of continuous data can be achieved by calculating standard deviations or employing medians. Chi-square test or rank sum test was used for univariate analysis, followed by Kaplan-Meier survival analysis to calculate the median survival time and 5-year cumulative survival. The association between variables and survival was assessed and explained using univariate and multivariate Cox proportional hazards regression models. The test level was α = 0.05.

### Availability of data and materials

The datasets used and/or analyzed during the current study are available from the corresponding author upon reasonable request.

## RESULTS

**Figure 1:**
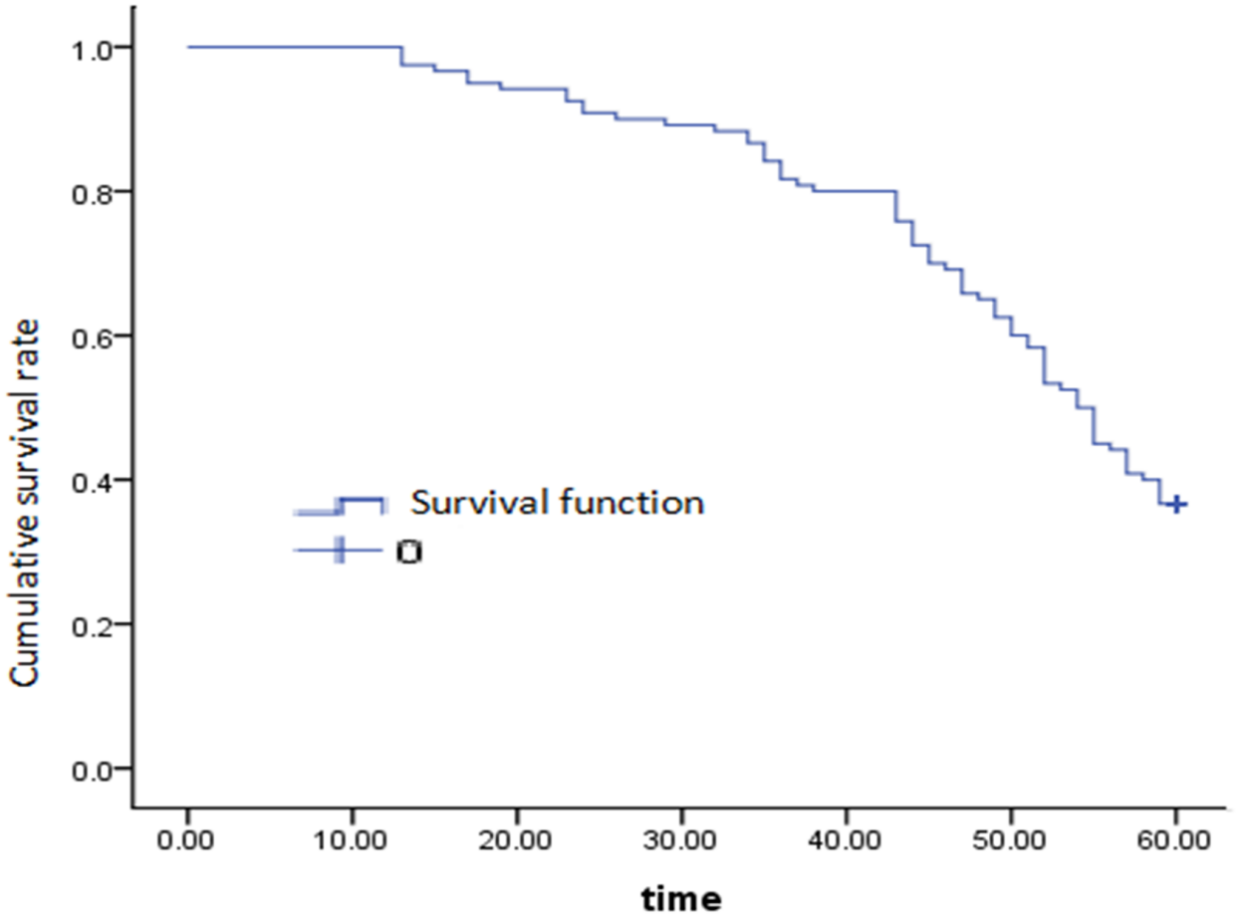
The 5-year overall survival curve of gastric cancer.

There were 5 cases of anastomotic leakage, 3 cases of postoperative anastomotic hemorrhage, 3 cases of pulmonary infection and 6 cases of incisional infection. All patients were cured and discharged. Patients were followed up for 0 to 60 months. Excluding the missing patients, the 5-year follow-up rate was 92.5%, the 5-year overall survival rate was 36.2% (Figure 1), and the median survival time was 53.0 ± 1.461 months.

### Analysis of patient survival

The results of K-M analysis revealed a negative correlation between the 5-year survival rate of patients and tumor location, differentiation degree, depth of invasion, regional lymph node metastasis, and postoperative tumor stage. (*P* < 0.05), while age, sex, CEA level, tumor size, lymphatic tumor thrombolus, and nerve invasion were not correlated with prognosis (*P* > 0.05). (Table 2).

**Table 2:** Results of univariate analysis affecting prognosis.

**Parameter**	**Case (*n* = 120)**	**Five-year survival rate %**	**χ^2^**	***P***
Age			2.433	0.119
<65 years	49	53.6		
≥65 years	71	37.3		
Sex			2.708	0.100
Female	56	53.6		
Male	64	40.6		
CEA level			0.306	0.580
Rise	44	46.2		
Normal	76	46.8		
Tumor location			10.673	0.033
Proximal gastric cancer	31	51.4		
Central Gastric cancer	21	47.1		
Distal gastric cancer	68	38.2		
Tumor differentiation			7.285	0.026
G1	15	59.1		
G2	43	41.9		
G3	62	34.8		
Invasion depth			13.260	0.001
T2	51	64.2		
T3/T4	69	32.8		
Tumor size			0.572	0.449
<5 cm	76	50.0		
≥5 cm	44	41.7		
Regional lymph nodes			13.533	0.004
N0	31	75.0		
N1	58	53.8		
N2	79	38.1		
N3	44	28.6		
Lymphatic carcinoma thrombus			0.058	0.810
No	75	50.0		
Yes	45	43.1		
Nerve invasion			1.152	0.283
No	87	43.7		
Yes	33	51.0		
pTNM stages			45.571	<0.001
Ib	29	86.4		
II	35	53.1		
III	56	12.1		
Nutritional status			8.254	0.078
Nutritional risk	4	39.5		
No nutritional risk	116	46.2		
Concomitant disease			0.996	0.352
Diabetes	33	52.8		
Hypertension	49	49.2		
Coronary heart disease	15	55.6		
ASA classification			2.447	0.115
ASA1	102	41.5		
ASA2	18	39.5		
Postoperative complication			3.554	0.096
Anastomotic leak	5	62.5		
Anastomotic hemorrhage	3	71.6		
Pulmonary infection	3	77.5		
Incision infection	6	69.4		

### Analysis of factors affecting survival

**Figure 2:**
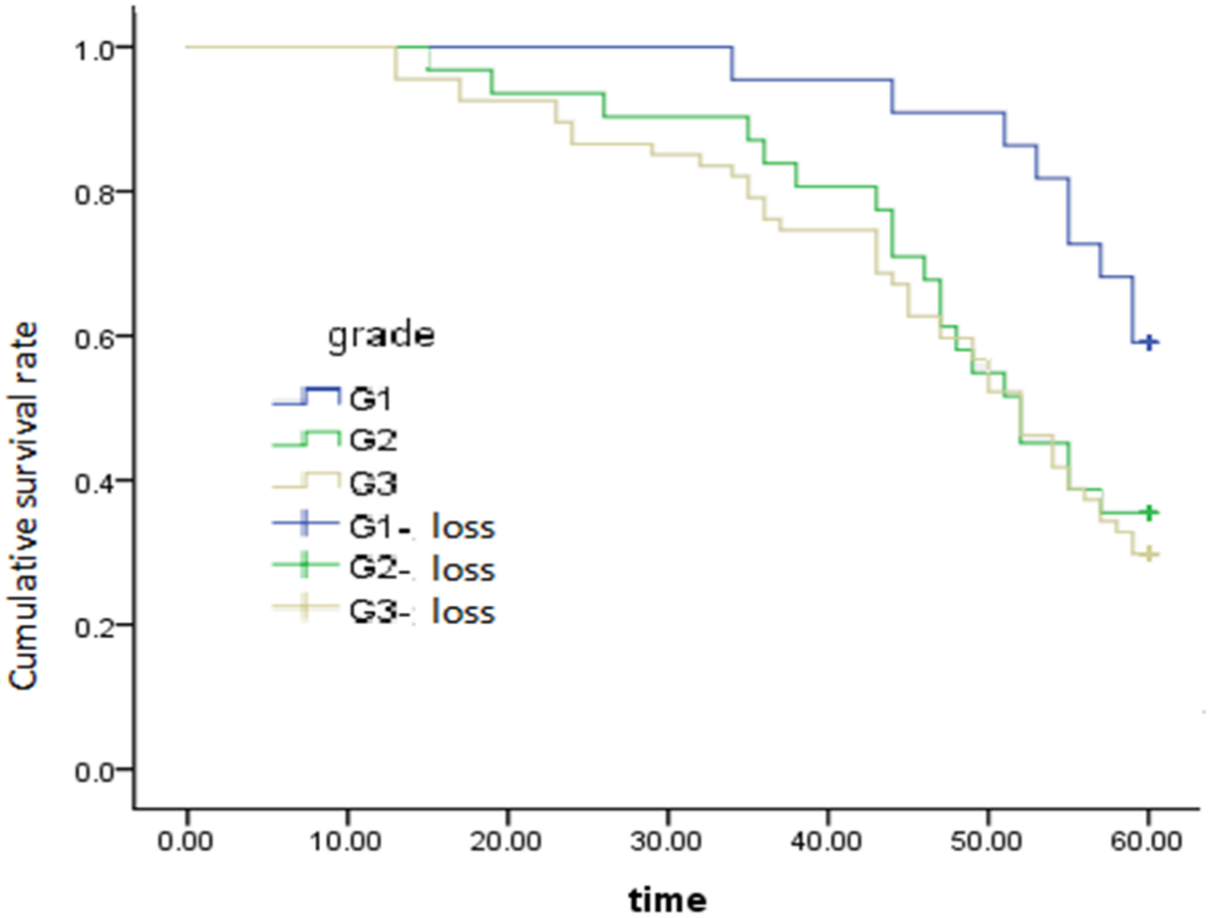
Survival curve of tumor differentiation on overall survival, *P* = 0.024.

**Figure 3:**
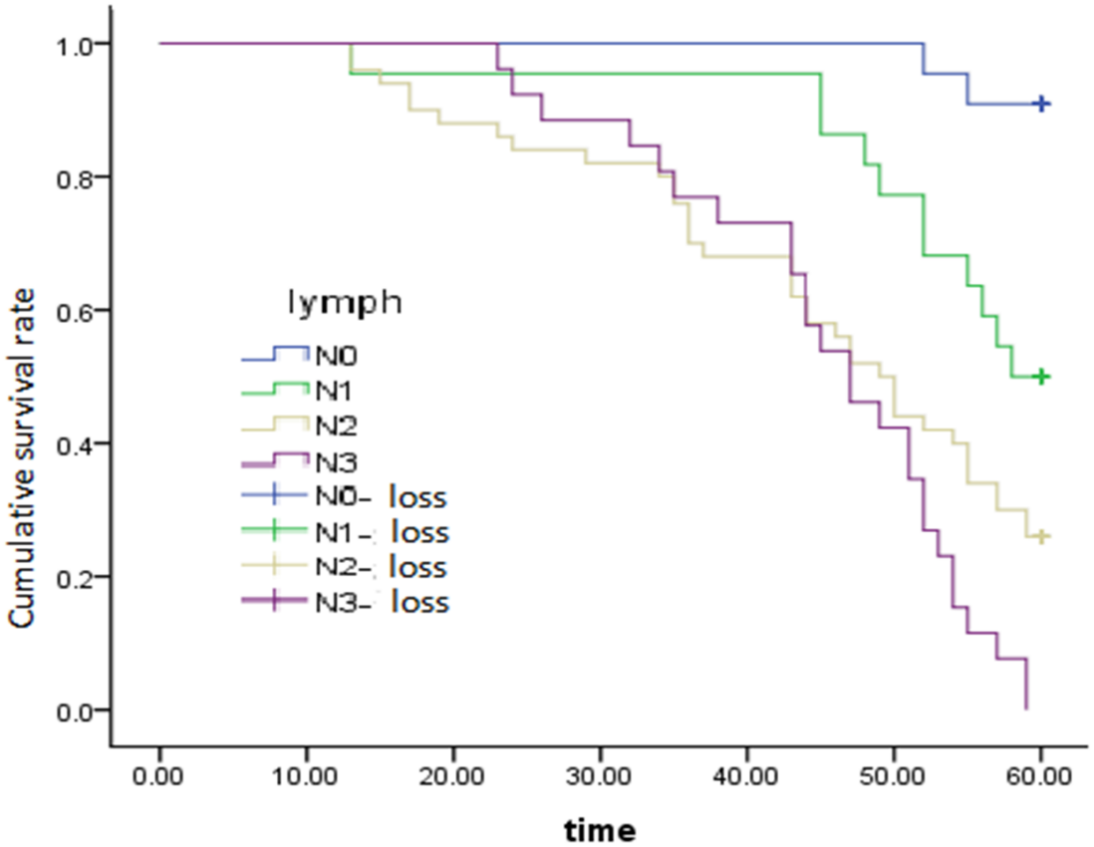
Survival curve of lymphatic and metastatic effects on overall survival, *P* = 0.005.

**Figure 4:**
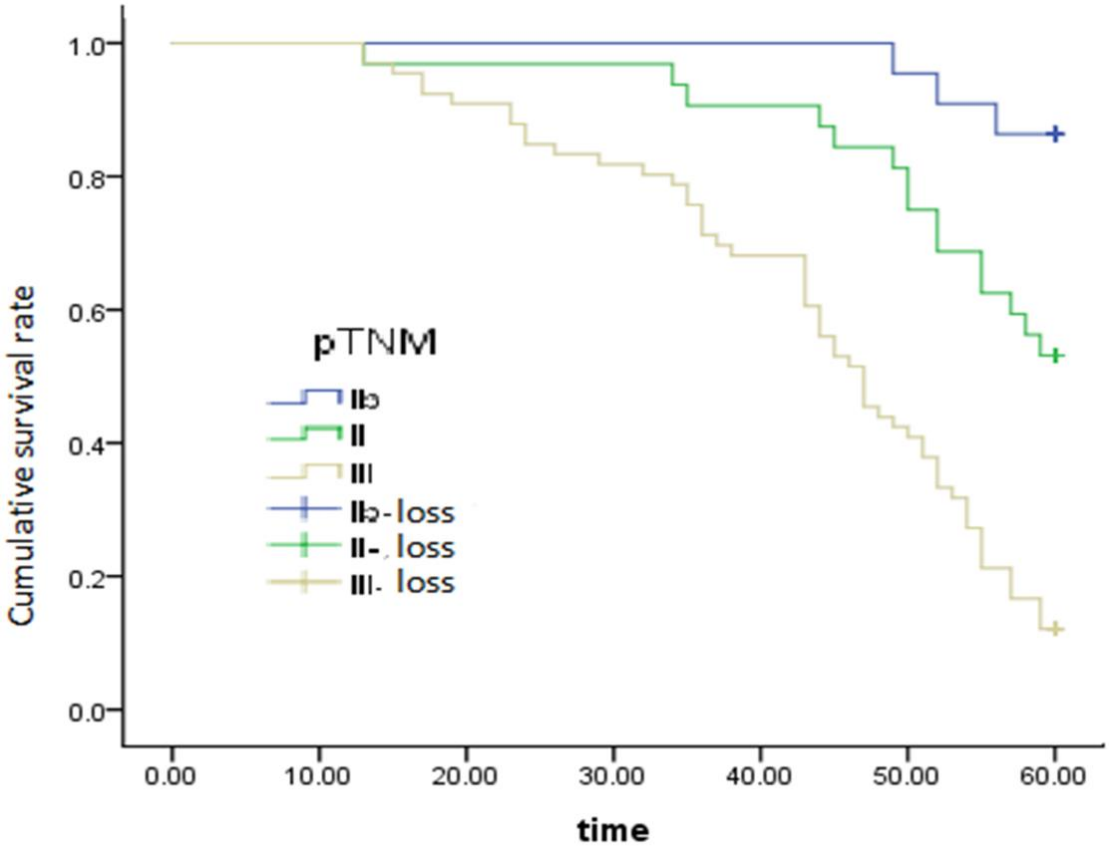
Graph of the effect of postoperative pathological staging on overall survival, *P* = 0.002.

After adjusting for and controlling for confounding variables, we screened out the independent predictors that significantly influence 5-year survival after radical surgery, including the degree of histological differentiation (HR = 1.441; 95% CI = 1.049–1.979, *P* = 0.024) (Figure 2), regional lymph node (HR = 1.626; 95% CI = 1.160–2.279, *P* = 0.005) (Figure 3), and pTNM stage (HR = 2.266; 95% CI =1.335–3.847, *P* = 0.002) (Figure 4), which were found to be independent risk factors for poor survival (Table 3). Tumor location (*P* = 0.191), invasion depth (*P* = 0.579) and tumor size (*P* = 0.324) were not found to be independent risk factors.

**Table 3:** Analysis results of multiple factors influencing prognosis.

**Parameter**	**B**	**SE**	**Wald**	**df**	**Sig.**	**Exp (B)**	**95% CI**	
							**Downside**	**Upside**
Tumor location	.181	.138	1.713	1	.191	.835	.637	1.094
Tumor differentiation	.365	.162	5.092	1	.024	1.441	1.049	1.979
Invasion depth	.147	.266	.307	1	.579	1.159	.688	1.951
Tumor size	.244	.248	.972	1	.324	.783	.482	1.273
Regional lymph nodes	.486	.172	7.955	1	.005	1.626	1.160	2.279
pTNM stages	.818	.270	9.172	1	.002	2.266	1.335	3.847

## DISCUSSION

### Analysis of the scope of lymph node dissection for advanced gastric cancer

The performance of lymph node dissection during surgical resection of gastric cancer not only plays a crucial role in staging, but also significantly impacts patient prognosis. Lymph node dissection during surgical resection of gastric cancer plays an important role not only in staging but also in patient prognosis. At present, the standard radical surgery for gastric cancer is D2 gastrectomy, which can be treated by open or endoscopic treatment. However, the development of this technique is limited to our hospital, and all patients in this group were treated by open D2 radical surgery. The purpose of lymph node dissection is to assess the extent of lymph node metastasis and enhance surgical outcomes. However, for patients with an advanced gastric cancer, the benefit of lymphadenectomy, in addition to D2 gastrectomy, remains controversial. According to the latest guidelines, if the tumor infiltrates the duodenum, it is recommended to perform resection of lymph nodes located posteriorly to the pancreatic head (Group 13), defined as D2+13. If Group 6 of the lymph nodes were found to have metastasis, 14v was removed; this group was called D2+14v. Therefore, in the tumor surgical guidelines of the Society of Oncology of Japan, Korea, and Europe, D2 lymphadenectomy is strongly recommended as the standard procedure for treating advanced gastric cancer. The National Comprehensive Cancer Center (NCCN) also recommended D1 or modified D2 lymph node dissection for advanced gastric cancer [6]. Advanced proximal gastric cancer may metastasize to the lymph nodes located at the splenic hilum, specifically No. 10 LN. Total gastrectomy combined with splenectomy is a method for complete resection of 10 groups of lymph nodes. However, splenectomy has many disadvantages for patients. A randomized controlled trial in Japan (JCOG0110) clearly showed that preventive splenectomy is not necessary unless the tumor has invaded the greater curvature [7]. Laparoscopic/robotic splenohilal resection with enhanced visualization has been developed that promises to replace preventive splenectomy with equal tumor outcomes and lower morbidity. The extensive dissection of lymph nodes directly impacts patient prognosis and increases the likelihood of postoperative complications, while also ensuring sufficient lymph node counts and a high rate of lymph node metastasis [8, 9]. For this reason, an appropriate scope of lymph node dissection can improve patient survival [10]. Lymphatic tracers can better guide lymph node dissection, and indocyanine green can significantly increase the number of D2 lymph node dissections without increasing complications. Indocyanine green fluorescence imaging can be used for routine lymphatic localization in laparoscopic gastrectomy, especially total gastrectomy [11]. Regional lymph node metastasis was an independent risk factor for survival in this cohort. Of course, lymph node metastasis, a factor affecting patient survival, is important [12]. As reported in the literature [13], there is a significant difference in survival even for patients with negative lymph nodes, indicating that the impact of other risk factors on survival cannot be ignored.

### Analysis of factors affecting the survival of gastric cancer patients

According to factor analysis, tumor location, size, and depth of invasion are not independent risk factors for prognosis, and more regional lymph node metastases are involved [14]. Although tumor location and invasion depth cannot be independent risk factors, they both affect the path of tumor metastasis to the lymph node and are potential correlation factors. During tumor progression, cancer cells migrate to regional lymph nodes under the action of chemokines. A positive regional lymph node affects the prognosis of patients; even if radical surgery is performed for early gastric cancer. In the process of tumor proliferation and invasion, cancer cells are influenced by chemokines to migrate towards regional lymph nodes. The presence of positive regional lymph nodes significantly impacts the prognosis of patients, even after undergoing radical surgery for early gastric cancer, if the lymph nodes are positive, the prognosis is relatively poor. As the number of lymph node metastases increased, the degree of invasion increased, the tumor heterogeneity increased, and the tumor evolution and survival rate decreased significantly. Kim et al. [15] analyzed 10050 patients with gastric cancer; N2-3 patients accounted for 29.2%, and the 5-year OS rates of N0, the N1, N2 and N3 patients were 93.3%, 78.1%, 64.3% and 33.7%, respectively. Lymph nodes should be dissected, with no less than 10 nodes in the T1 stage and no less than 16 nodes in other T stages, to better determine the status of lymph node metastasis and its impact on long-term survival [16–18]. In this study, the regional lymph node status was found to be an independent risk factor for gastric adenocarcinoma survival. Compared with the 6th edition, the 7th edition of the TNM classification system is more reliable and accurate at classifying the number of metastatic lymph nodes to predict OS after radical gastrectomy [19]. The degree of tumor differentiation is an observation index for endoscopic treatment of early cancer, and the frequency of lymph node metastasis and submucosal invasion of poorly differentiated adenocarcinoma is high [20]. In this cohort, the 5-year survival rate of poorly differentiated patients was 34.8%. A poorly differentiated tumor and an increased probability of regional lymph node metastasis were found to be independent prognostic risk factors. The stage of cancer is the most important independent prognostic factor [21–24]. The Cox model was used in some studies to show that the risk of death in stage III patients is 2.82 times that in stage II patients, and that in stage IV patients is 3.29 times that in stage II patients [21]. The presence of PTNM serves as a significant independent prognostic indicator for patients with gastric cancer patients [25, 26] and can forecast patients’ prognosis and offer treatment recommendations. The prognosis of patients varied significantly across different disease stages. [27]. The relationship between the prognosis of 10,050 patients with stomach cancer and various stages was analyzed by Kim et al. [15]. According to the AJCC staging system of the 7th edition [28], stage III patients accounted for 25% of the patients. The 5-year OS rates of patients with stage Ia, Ib, IIa, IIb, IIIa, IIIb, IIIc and IV disease were 96.1%, 93.5%, 84.9%, 76.1%, 66.7%, 43.8%, 24.9% and 10.1%, respectively. According to our clinical data analysis, pTNM stage had been confirmed as an independent risk factor in gastric cancer, and with the advanced stage, the clinical prognosis was correspondingly poor. In this cohort, the 5-year survival of stage Ib patients was significantly greater than that of stage II and III patients. Therefore, studying how to detect gastric cancer early is highly important for its prevention and treatment.

The present study is a retrospective analysis conducted at a single research center, which lacks data analysis of multi-center bulk medical records, and has certain research defects, which may affect the results of multi-factor analysis. More clinical data will be included in later studies.

In conclusion, the differentiation of tumors, the metastasis to regional lymph nodes and postoperative pathological stage were found to be independent risk factors for 5-year overall survival in patients with advanced gastric cancer. Standard and reasonable lymph node dissection and accurate postoperative pathological staging provide important support for the prognosis of advanced gastric cancer patients.
